# Correlative Analysis of the Dimensional Properties of Bipyramidal Titania Nanoparticles by Complementing Electron Microscopy with Other Methods

**DOI:** 10.3390/nano11123359

**Published:** 2021-12-10

**Authors:** Loïc Crouzier, Nicolas Feltin, Alexandra Delvallée, Francesco Pellegrino, Valter Maurino, Grzegorz Cios, Tomasz Tokarski, Christoph Salzmann, Jérôme Deumer, Christian Gollwitzer, Vasile-Dan Hodoroaba

**Affiliations:** 1 Laboratoire National de Métrologie et d’Essais (LNE), 29 Avenue Roger Hennequin, CEDEX, 78197 Trappes, France; nicolas.feltin@lne.fr (N.F.); Alexandra.Delvallee@lne.fr (A.D.); 2 Dipartimento di Chimica and NIS Inter-Department Centre, University of Torino, Via P. Giuria 7, 10125 Torino, Italy; francesco.pellegrino@unito.it (F.P.); valter.maurino@unito.it (V.M.); 3 Academic Centre for Materials and Nanotechnology, AGH University of Science and Technology, Mickiewicza 30, 30-059 Krakow, Poland; ciosu@agh.edu.pl (G.C.); tokarski@agh.edu.pl (T.T.); 4 Federal Institute for Materials Research and Testing (BAM), Unter den Eichen 44-46, 12205 Berlin, Germany; ch.salzmann@web.de (C.S.); dan.hodoroaba@bam.de (V.-D.H.); 5 Physikalisch-Technische Bundesanstalt (PTB), Abbestraße 2–12, 10587 Berlin, Germany; jerome.deumer@ptb.de (J.D.); christian.gollwitzer@ptb.de (C.G.)

**Keywords:** nanoparticle, complex-shape, bipyramid, electron microscopy, atomic force microscopy, size measurements, TKD, STEM-in-SEM, SAXS, nanoparticle concentration, correlative analysis

## Abstract

In this paper, the accurate determination of the size and size distribution of bipyramidal anatase nanoparticles (NPs) after deposition as single particles on a silicon substrate by correlative Scanning Electron Microscopy (SEM) with Atomic Force Microscopy (AFM) analysis is described as a new measurement procedure for metrological purposes. The knowledge of the exact orientation of the NPs is a crucial step in extracting the real 3D dimensions of the particles. Two approaches are proposed to determine the geometrical orientation of individual nano-bipyramides: (i) AFM profiling along the long bipyramid axis and (ii) stage tilting followed by SEM imaging. Furthermore, a recently developed method, Transmission Kikuchi Diffraction (TKD), which needs preparation of the crystalline NPs on electron-transparent substrates such as TEM grids, has been tested with respect to its capability of identifying the geometrical orientation of the individual NPs. With the NPs prepared homogeneously on a TEM grid, the transmission mode in a SEM, i.e., STEM-in-SEM (or T-SEM), can be also applied to extract accurate projection dimensions of the nanoparticles from the same sample area as that analysed by SEM, TKD and possibly AFM. Finally, Small Angle X-ray Scattering (SAXS) can be used as an ensemble technique able to measure the NPs in liquid suspension and, with ab-initio knowledge of the NP shape from the descriptive imaging techniques, to provide traceable NP size distribution and particle concentration.

## 1. Introduction

According to the ISO definition [1], a nano-object is an object with one or more external dimensions in nanoscale (NanoED), i.e., ranging from 1 to 100 nm: (i) 3 NanoED = nanoparticles (NP), (ii) 2 NanoED = nanofibers, and (iii) only 1 NanoED = nanoplates. Many techniques can then be used to measure the external dimensions of a nano-object. Thus, microscopy techniques such as the Electron Microscopy (EM) or the Atomic Force Microscopy (AFM) enable to visualize the measured objects and are directly traceable to the SI meter. However, these techniques alone are limited to a 1D (AFM) or 2D (EM) measurement of the nano-objects with a controlled uncertainty and only allow to routinely image a tiny fraction of the entire population.

The EM or AFM are widely-used techniques for the morphological analysis of a nano-object, but measuring three external dimensions remains a challenging task and the reliability of the measurement may depend on the orientation of the particles deposited on the sample substrate. An image generated by EM can provide only dimensional data in the XY plane of the image (parallel to the scanning plane). Depending on the particular type of electron signal coming from the sample, the various types of electron microscopies such as SEM (Scanning Electron Microscopy), TEM (Transmission Electron Microscopy), STEM-in-SEM (Scanning Electron Microscopy in the Transmission Mode) associated with different types of detection offer more or less qualitative information on the topography of the sample/nanoparticles surface, but no quantitative information is given along Z-axis. Therefore, in the case of a nano-object with three different external dimensions, EM appears unsuitable unless 3D electron tomography is applied. Three-dimensional image reconstruction with a SEM is basically possible either by tilting the sample or using collection of electrons under different angles, however, not at the nanometer scale. On the other hand, in principle, the use of AFM would be sufficient to measure a nano-object deposited on a substrate in three dimensions of space. However, the tip/object convolution occurring when the size of the tip and the object to be imaged are of the same order of magnitude impacts negatively the reliability of the lateral dimension measurements of the nano-object [2]. In contrast, the height of the object is not affected by this convolution and can be measured by AFM with an uncertainty around 1 nm [3,4].

Ensemble techniques such as small angle X-ray scattering (SAXS), enable the entire population to be measured without need of significant effort in sample preparation. However, as these methods are indirect, the spherical shape approximation is generally applied [5]. Thus, for non-spherical particles, this approximation leads to significant errors associated with the dimensional measurements or failure to fit the model to the data. However, models could be used to determine these dimensions provided that input data (shape, aspect ratio) is available.

Studies have already been performed to show that the comparison of EM, AFM and SAXS techniques on near-spherical reference NP give consistent results [6]. Moreover, a recent study performed on near-spherical reference NP [7] shows that the combination of AFM with EM enables a good understanding of the particle shape. However, to our knowledge, no studies have been reported to obtain a complete dimensional characterization in 3D for the case of particles with a more complex shape.

In this study, we propose to combine EM and AFM to achieve a complete 3D characterization of complex shape NP and get reliable measurements despite various orientations of the NP on the substrate. Thus, one aim is to determine the external dimensions as well as the shape of the particles on a few hundred objects. The same sample in liquid suspension form will be then measured by SAXS to get comprehensive information which is independent of the NP orientation. In order to evaluate and interpret the SAXS signal, a specific model needs to be considered. This model uses as input data the shape of the particles as determined by microscopy in order to make the SAXS measurements more reliable. In this way, a new tiered approach could be suggested to laboratories involved in the size and shape measurements of complex nanoparticle. If the nano-object has a simple shape with three equal dimensions (for instance perfect sphere or cube), the determination of a single parameter is needed to characterize the size of the nano-object which can be easily deduced either from measurements of height by AFM or lateral dimensions (i.e., diameter or cube edge) by EM.

In the case of a nano-object with two similar external dimensions (for instance, spheroidal NP, acicular NP, nanotubes, nanorods, etc.), both characteristic parameters (semi-axes and length) can be directly determined by EM only if the major axis of the nano-object is oriented parallel to the sample surface. Consequently, the reliability of the measurements depends on the geometrical orientation of the nano-objects deposited on the substrate. A preferred orientation may be related to the value of the ratio between major and minor axes, but also to the surface chemistry of the nanoparticles as well as of the substrate or to sample preparation (rests of solvent or number of particles per unit area of substrate).

In the case of complex shape particles that do not have a preferential orientation on the substrate, it is necessary to emphasize that combined microscopy techniques enable characteristic dimensions to be measured by taking into account their orientation on the substrate. Furthermore, we want to demonstrate that SAXS is a simpler alternative method to be implemented for this type of object, provided that the NP morphology is known a priori.

In this paper, we propose analysis of a bi-pyramidal TiO_2_ NP population with EM and AFM, with the purpose to also feed the SAXS model. Several solutions will be implemented to determine the geometrical orientation of the bi-pyramids on the substrate. These will include individual measurement approaches such as the analysis of AFM profiles or the EM observation of the same particle at different stage tilts. The Transmission Kikuchi Diffraction (TKD) will also be used to obtain more integral information on the preferential orientation of the NP. Depending on the orientation, the characteristic NP parameters (length, width and aspect ratio) will be calculated. The aspect ratio will be included in the SAXS model. Based on this, the external dimensions of the bipyramids will be evaluated and compared to the ones obtained by microscopy.

## 2. Materials and Methods

### 2.1. The TiO_2_ Nanoparticles

**Figure 1:**
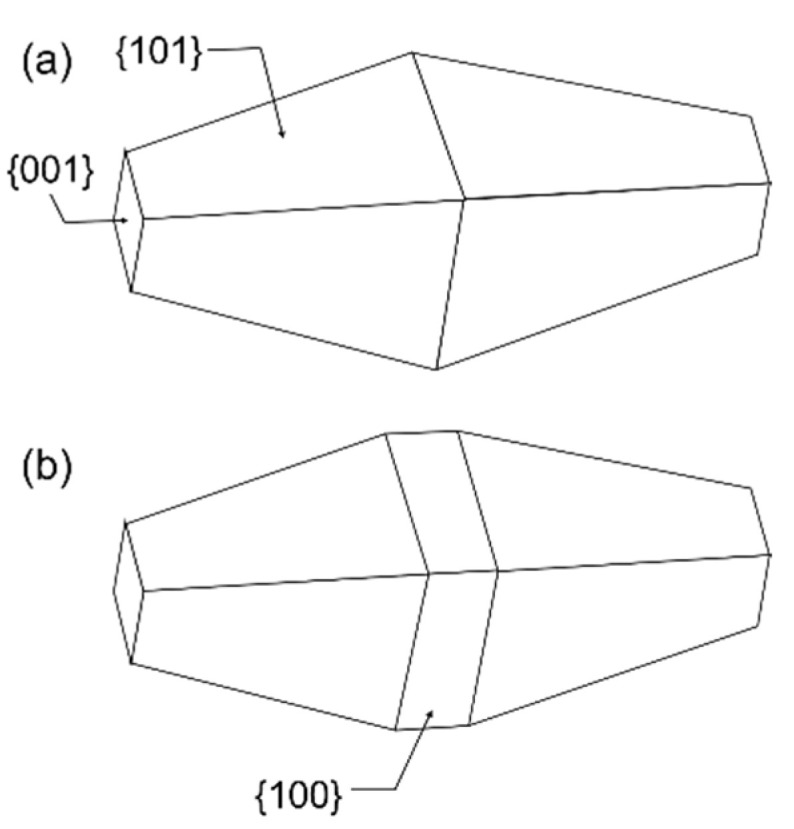
(**a**) TiO_2_ (anatase) nanoparticles as tetragonal bipyramids with height equivalent {101} faces. The truncation at the top of each pyramid gives rise to the formation of the {001} faces, (**b**) TiO_2_ (anatase) tetragonal bipyramids with height equivalent {101} faces and four {100} faces on the edge of square base.

Anatase TiO_2_ nanoparticles with shape-controlled bypiramidal morphology (TiO_2_ NPs) were synthesized by University of Turin under the frame of the EMPIR nPSize project (improved traceability chain of nanoparticle size measurements) following the protocol described by Mino and al. [8]. Triethanolamine (TEA, reagent grade 98%, Merck, Darmstadt, Germany) was used as a surface ligand in the suspensions [9]. According to the manufacturer, the nominal characteristics dimensions of these particles are 60 nm for the length and 40 nm for the width. The theoretical shape of the bi-pyramids is presented in Figure 1.

According to the Wulff theory, based on the assumption that the minimization of the surface energy leads to the optimal structure of the crystal surface, the most stable shape of the anatase crystallites corresponds to a slightly truncated tetragonal bipyramid, with height {101} isosceles trapezoidal faces as well as two {001} upper faces at each end and four {100} faces on the edge of square base (Figure 1).

**Figure 2:**
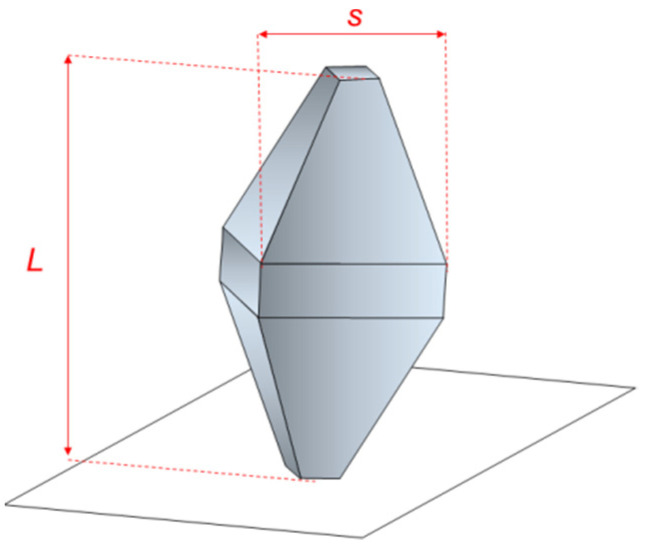
Schematic representation of a truncated bi-pyramidal NP with the following characteristic parameters: (i) L, length, (ii) s, side of the square base, (iii) major axis truncation and (iv) minor axis truncation.

The main characteristic dimensional parameters describing a truncated bi-pyramidal shape are *L* and *s*, length and square base side, respectively, and the eventual truncations of the pyramids along and perpendicular to their major axis (Figure 2).

**Figure 3:**
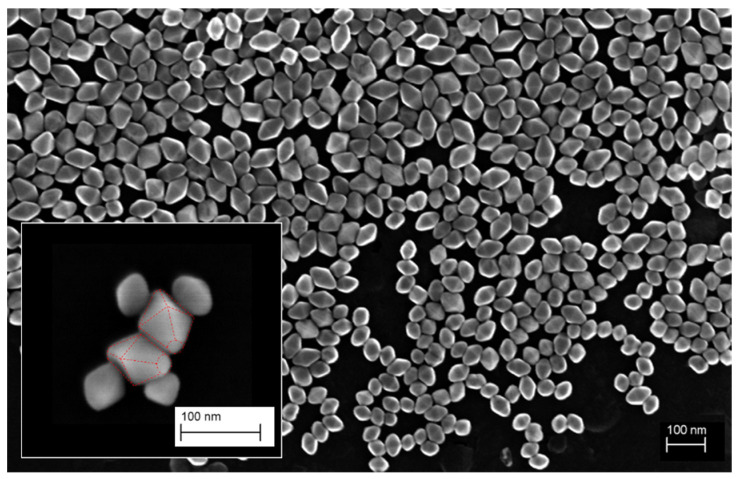
Twenty kilovolt InLens SEM images of TiO_2_ NPs deposited on a carbon TEM grid. Insert: Representation of bipyramid geometry on particles imaged by 3 kV InLens SEM on a silicon substrate.

One example of the bi-pyramids after deposition on a silicon substrate is visualized in Figure 3. The arbitrary orientation of the nanoparticles is obvious. The corresponding STEM-in-SEM image is given in Figure S1. There one can observe that no clear statement on the exact orientation of the particles can be made. Further advantages and disadvantages of the two correlative imaging electron microscopy modes are explained in detail in Section 4.2.1. However, to be noticed this is not the general case for routine analysis at an SEM, and dedicated, separate analyses are necessary.

For routine analysis, the TiO_2_ NPs were deposited on a silicon substrate by using a spin-coater to prevent any agglomeration process of the NPs [10]. Prior to deposition, the substrate was functionalized with Poly-L-Lysine (PLL, SERVA Electrophoresis GmbH, Duisburg, Germany) in order to enhance the adhesion of the nanoparticles [11]. We made sure that the PLL did not affect significantly the result of the AFM measurement. Indeed, the roughness (*S_q_*) of the substrate surface after deposition was evaluated at 1.0 nm.

Regarding STEM-in-SEM and TKD analyses, NPs were deposited on a carbon grid using spin coating. The Table 1 gathers the information on the NP deposition method for each of the microscopy techniques.

### 2.2. Scanning Electron Microscopy (SEM)

The SEM measurements were performed with a Zeiss-Ultra+ field-emission gun (FEG) microscope (Zeiss, Oberkochen, Germany) equipped with a GEMINI optical column and an In-Lens secondary electron detector located within the column. The setting parameters were held constant over study period. The electron high tension (EHT, accelerating voltage) corresponding to the primary electron energy and the working distance (WD) were set at 3 kV and 3 mm, respectively. The FEG-SEM resolution was about 1.7 nm (manufacturer specifications) for EHT = 1 kV and 1.0 nm at 15 kV (for 2 mm WD). The pixel size was 1.4 nm with a 28.4 s total cycle time for recording an image. The values assigned for contrast and brightness were 31.4 and 49%, respectively.

### 2.3. Atomic Force Microscopy (AFM)

The AFM is a Veeco Nanoman V (Bruker, MA, USA) equipped with an accurate three-axis scanner operating under closed-loop control (hybrid XYZ-scanner with a range of 90 μm × 90 μm × 8 μm). All measurements were carried out with OTESPA-R3 (Bruker, MA, USA) probes (roughly 7 nm nominal radius of curvature of the tip) using the tapping mode. The nominal stiffness of the cantilever is 42 N/m and its resonance frequency is 300 kHz. The tip oscillation amplitude was always about 40 nm. The amplitude setpoint was set near the free amplitude (80%) value to prevent too strong interactions with the sample nanoparticles which could lead to their displacements. On all images, the pixel size was close to 5.0 nm. The scan speed was kept to 4 μm/s with constant feedback parameters (0.8 for integral gain and 10 for the proportional one).

### 2.4. Transmission Kikuchi Diffraction (TKD)

The TKD method is a relatively recent development evolved from the conventional Electron Backscatter Diffraction (EBSD), and is applied on samples which are electron-transparent in the transmission mode. The reduced interaction volume in the sample results in a spatial resolution of TKD which is below 10 nm and, hence, well-suited for the analysis of the crystallography (orientation and phase) of individual NPs [12,13]. The technique has a high analysis throughput, providing results relying on a large number of NPs. The TiO_2_ NPs were deposited on a carbon TEM grid. The grid was placed on a −60° pre-tilted TKD sample holder in a Versa 3D field emission gun SEM (FEI, Hillsborough, OR, USA) equipped with a Symmetry S2 electron backscatter diffraction detector (Oxford Instruments NanoAnalysis, High Wycombe, UK). The pre-tilted sample holder together with the SEM stage tilt of 40° resulted in a total sample tilt of −20°, which is typical for off-axis TKD measurements. The SEM was operated at an accelerating voltage of 30 kV and beam currents of about 16 nA. The TKD maps were collected at Speed 2 (156 × 128 pixels) mode and Gain 2 of the detector, resulting in 372 patterns per second at a step size of 5 nm. Raw patterns were corrected by auto and static correction. Static background was collected in grid area densely covered with NPs. The refined accuracy [14] indexing routine was used in the available Aztec software (Oxford Instruments NanoAnalysis, High Wycombe, UK) for indexing patterns with increased angular resolution. For the presentation of the available quality of Kikuchi diffraction from TiO_2_ NPs, full camera resolution pattern is presented in the Figure S2a and indexed in Figure S2b. The crystallographic orientation analysis was performed using the AztecCrystal version 2.1 software package (Oxford Instruments NanoAnalysis, High Wycombe, UK).

### 2.5. Transmission SEM (STEM-in-SEM)

Once the TiO_2_ NPs are deposited as isolated particles on an electron-transparent substrate, i.e., a TEM grid like for the TKD analysis above, the transmission mode in an SEM (STEM-in-SEM, or T-SEM) can be applied to analyze the projection image of the NPs and determine their lateral dimensions. The transmission contrast responsible for the determination of the lateral dimensions of NPs by STEM-in-SEM is superior to that of SEM [15,16]. Thus, the particle boundaries are better defined than in SEM, and STEM-in-SEM is well-suited for the determination of the lateral dimensions of the NPs with high accuracy. Two types of STEM-in-SEM imaging are available: (i) by using a so-called STEM detector placed under the TEM grid with the NPs, and (ii) by using a dedicated transmission sample holder which forces the transmitted electrons to be collected by the already available Everhart-Thornley electron detector [16]. In the current study, the latter version has been used in conjunction with a Zeiss SEM of type Supra 40 equipped with a Schottky field-emitter (Zeiss, Oberkochen, Germany). Remarkably is the possibility of correlative electron microscopy of the same field-of-view with the same NPs deposited on an electron-transparent substrate by the two options: (i) SEM via the InLens detector (top-view imaging) sensitive to the surface morphology, and (ii) STEM-in-SEM with its material (mass-thickness) contrast sensitive to lateral dimensions, due to the almost binary character of the transmission image.

A quantitative systematic evaluation of the entire sequence of correlative imaging methods AFM/SEM/TKD/STEM-in-SEM has not been carried out on the same NPs as deposited on the same (type of) substrate due to the availability of different method combinations at different partners/authors. Note that—to our knowledge—such an instrumental possibility is not available, so that at present only pairs of methods can be correlated.

### 2.6. Small Angle X-ray Scattering (SAXS)

The TiO_2_ NPs have been analyzed by SAXS in liquid suspension. SAXS is a non-destructive ensemble method which can characterize NP suspensions and is based on the forward scattering of X-rays under small angles from the incident beam direction. Among other factors, the measured SAXS signal depends on the particles’ shape, size distribution, electron density contrast between particles and suspension medium, and the number concentration of the particles. The size distribution and number concentration can be extracted from the measured scattering pattern expressed in absolute units by fitting the data under the assumption of an adequate particle shape, which is mathematically described by the corresponding form factor.

The SAXS experiments were conducted at the four-crystal monochromator beamline of the PTB laboratory at the synchrotron radiation facility BESSY II at a photon energy of 8 keV [17]. The particle suspensions were enclosed in rectangular capillaries made of borosilicate glass (Hilgenberg GmbH, Malsfeld, Germany) with a homogeneous thickness along the capillaries’ vertical axis and sealed by welding for the measurement in ultra-high vacuum. In order to determine the thickness of the capillary, Fluorinert FC-3283 was filled into the capillary at the bottom. This fluid does not mix with the aqueous particle suspension filled on top of it, and the thickness of the capillary was determined from the transmission through the Fluorinert layer with a known attenuation coefficient [18]. SAXS patterns of the particles were subsequently measured at different positions along the capillary and the curves were averaged over a selection of the curves where the influence of the sedimentation of the particles was negligible.

The averaged SAXS curve was then fitted with a form factor for truncated bipyramids, which was numerically computed from Debye’s scattering equation using the CDEF method [19].

## 3. Instrument Calibration and Measurement Traceability

### 3.1. AFM Calibration

AFM Z calibration was performed by using the reference structure P_900_H_60_ presented in [7]. The geometry is a 3D grating with a nominal X/Y pitch equal to 900 nm and a step height of 60 nm. The P_900_H_60_ was calibrated beforehand using the LNE’s metrological AFM (mAFM) [20]. mAFM is the French national reference instrument ensuring the dimensional measurement traceability at the nanoscale level.

### 3.2. SEM Calibration

The image pixel size calibrated against a transfer standard by measuring the pitch in X and Y directions [21] using a Fourier transform. The used standard was the P_900_H_60_ presented in [7]. Measuring P_900_H_60_ pitch by means of the mAFM and SEM makes it possible to define a correction factor, *c*_P900H60_:

*c*_P900H60_ = *d*_SEM_/*d*_mAFM_

with *d*_SEM_ and *d*_mAFM_ denoting the pitch measurement carried out with SEM and mAFM, respectively.

Ten different P_900_H_60_ surfaces were imaged by SEM. For all images, the pitch was evaluated by Fourier transform. Standard deviation of the measurements between the 10 surfaces makes it possible to establish the uncertainty associated with the measurement of the P_900_H_60_ pitch by SEM.

**Figure 4:**
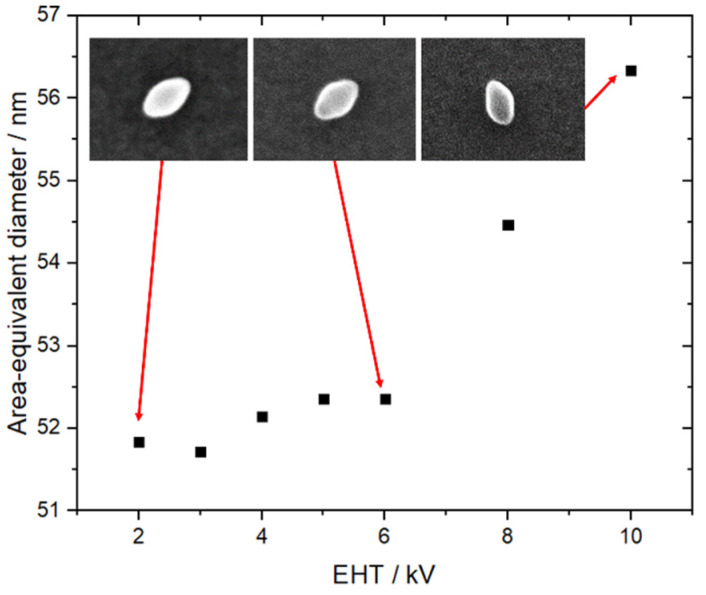
Area-equivalent diameter of a set of TiO_2_ nanoparticles as a function of the accelerating voltage ranging from 2 to 10 kV.

Then, SEM measurements were carried out on the TiO_2_ NPs population of 200 particles by varying the accelerating voltage in a range from 2 to 10 kV. The area-equivalent diameters, *D*_AE_, of a set of TiO_2_ NPs as a function of the accelerating voltage are reported in Figure 4.

The curve exhibits a minimum value of *D*_AE_ corresponding to the most likely value of the size, as demonstrated in a previous study [21]. This value is related to a 3 kV EHT. The list of setup parameters is given in Table 2.

## 4. Results and Discussion

### 4.1. Determination of the TiO_2_ NPs Geometrical Orientation

The measurement challenge lies on the ability of measuring the dimensional properties of the TiO_2_ NPs population by combining EM with AFM with TKD and SAXS.

**Figure 5:**
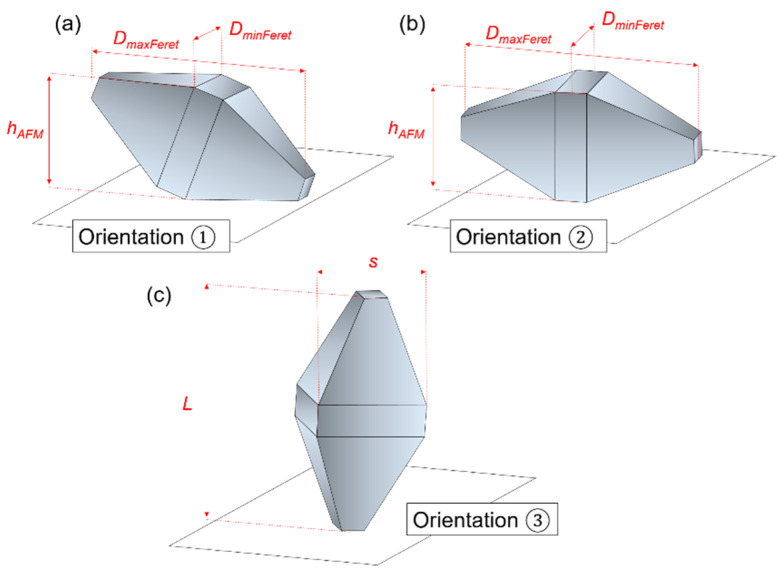
The three basic possible geometrical orientations for TiO_2_ NPs in contact with the silicon substrate for the ideal case that the particles are isolated from each other. (**a**) NP with one of its {101} faces in complete contact with the substrate, (**b**) NP rests on one side of its {100} square base and (**c**) NP in standing position. In orientation 3 the parameters L (length) and s (square base side) are indicated.

Regarding microscopy techniques, the different possible orientations of particles on the substrate make direct measurement by SEM difficult, as shown in the image reported in Figure 3 (left). Indeed, the complex shape leads to three possible basic orientations represented in Figure 5, as long as the TiO_2_ NPs are deposited as isolated/non-overlapping particles on the substrate. Touching particles will likely result also in geometrical orientations other than the three ones in Figure 5.

**Figure 6:**
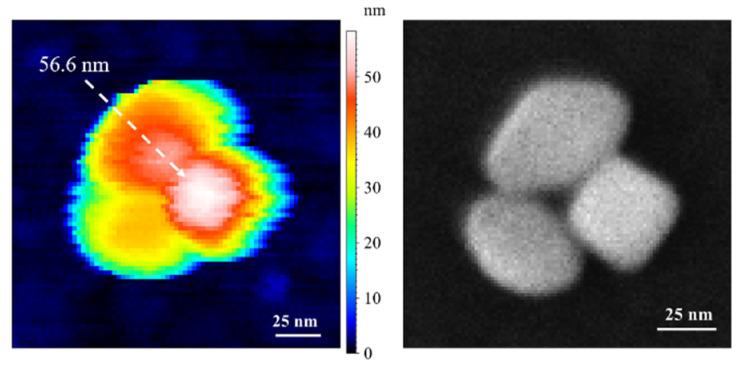
(**Left**): AFM image of 3 agglomerated TiO_2_ NPs. The arrow indicates the TiO_2_ NPs in standing orientation (③) with the AFM height of 56.6 nm, and (**right**): correlative SEM image of the same agglomerated TiO_2_-NPs with a measured *D*_fmin_ for the right particle in orientation ③ of 40.9 nm.

The schematic view of the orientation ① corresponds to a TiO_2_ NP with one of its {101} faces in complete contact with the substrate. As shown in this Figure, the length L cannot be directly determined only by the measurement of maximum Feret diameter (*D*_fmax_) in a projection image. However, the square base side is easily measurable through the minimum Feret diameter (*D*_fmin_). In contrast, in cases where the TiO_2_ NP rests on one side of its {100} square base, i.e., the orientation ②, both parameters *D*_fmax_ and *D*_fmin_ can be measured by SEM. Finally, the standing position, schematized as ③, was only observed within agglomerates as shown in Figure 6, see the right square-like particle, which is measured as the highest with AFM.

It is obvious that for isolated deposited TiO_2_ NPs, the two most likely orientations are ① and ②. In this study, we primarily aim to demonstrate that the combination of AFM and SEM techniques enables to determine the *L* and *s* parameters of a TiO_2_ NPs population.

#### 4.1.1. AFM—Profile along the Long NP Axis

**Figure 7:**
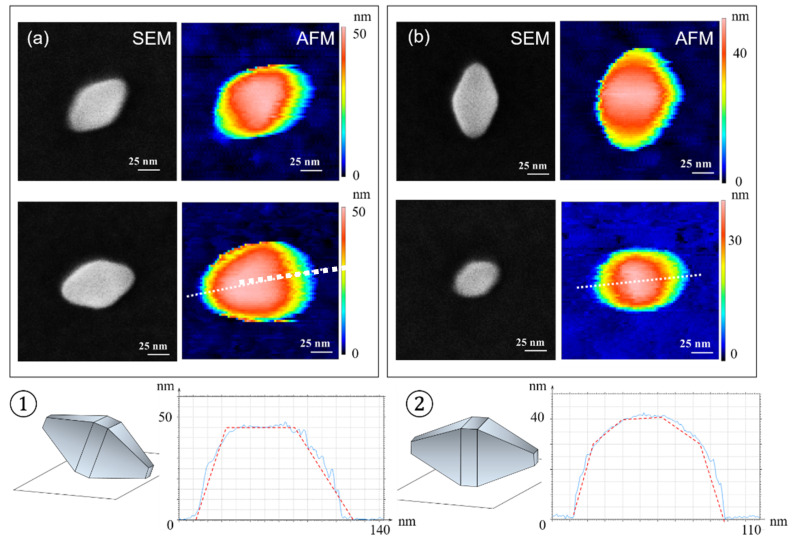
(**a**) AFM (colored) and SEM (black-and-white) image examples of TiO_2_ NPs with the orientations ① and (**b**) AFM (colored) and SEM (black-and-white) image examples of TiO_2_ NPs with the orientations ②. For each orientation, an AFM profile along the major axis of the TiO_2_ NPs (as shown by dotted lines) is displayed at the bottom.

Regarding all nano-bipyramids oriented according to ①, a triangular feature is clearly observed at the top of the TiO_2_ NPs in AFM images (Figure 7a). This triangle corresponds to the {101} face as the highest structure scanned by the AFM tip. The AFM profile given at the bottom of the Figure 7a and taken along the major axis of the TiO_2_ NPs (bottom left) in the orientation ① is asymmetric with a plateau at the top. Firstly, an abrupt slope is observed related to the bipyramid part not touching the substrate and a more gradual slope on the side in contact with the surface. In between both, the plateau is representing the {101} face of the TiO_2_ NPs.

In contrast, in the case of the orientation ②, both AFM images and profiles across the TiO_2_ NPs major axis are symmetric. Furthermore, the upper part of the particle is flat, narrow and rectangular. This demonstrates that the structure is similar to the one reported in Figure 7b with the presence of four {100} faces on the edge of square base.

**Figure 8:**
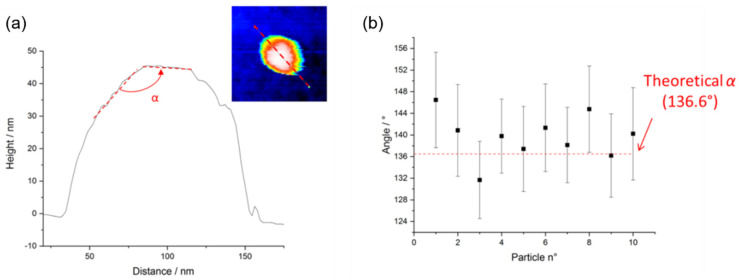
(**a**) Method for the estimation of the α angle from AFM profiles measured along the major axis of a TiO_2_ NP deposited on a silicon substrate. (**b**) α values determined from a set of 10 TiO_2_ NPs imaged by AFM represented together with the theoretical value.

Furthermore, the AFM measurements allowed us to estimate the α angle between two adjacent {101} faces, see Figure 8. This angle is theoretically equal to 136.6°. We can notice that the measured angles are very close to the value of the theoretical angle (below 7%).

#### 4.1.2. SEM—Stepwise Stage Tilt and Imaging

**Figure 9:**
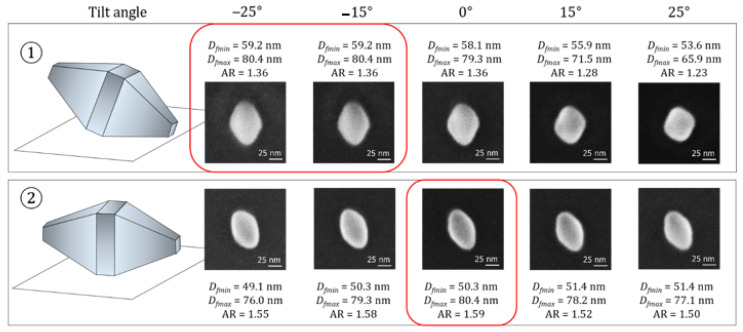
Changes in the 3 kV SEM images of TiO_2_ NPs performed after tilting the stage of the instrument between –25° and +25° in five steps. An example for each orientation ① and ② is given.

A second method can be used for the determination of the TiO_2_ NPs orientation through the SEM technique by tilting the stage of the instrument. The tilt angle was varied in five steps from −25 to 25°. Only NPs whose major axis is perpendicular to the tilt direction were studied. Two examples with the two different orientations ① and ② are presented in Figure 9.

Monitoring the angle, in which the *D*_fmax_ value reaches a maximum, allows us to identify the two different geometric orientations of the imaged TiO_2_ NP deposited on the substrate. When the maximum value of *D*_fmin_ is measured at 0° tilt, the particle is oriented according to ②. In the opposite case, this implies that one face of the pyramid is in parallel contact with the silicon surface (i.e., orientation ①). The deviations from the *D*_fmax_ value at 0° were measured from 1.0 to 5.2 nm. One can observe that the evolution of *D*_fmax_ as function of tilt angle *α* is less pronounced than the law *D*_fmax_ = *L.cos(α)*. Thus, it is not possible to predict the theoretical length *L* of the TiO_2_ NPs with orientation ① as a function of *D*_fmax_ and their theoretical angle of inclination.

Obviously, the maximum value reached by *D*_fmax_ corresponds exactly to the desired parameter, *L*, the length of the TiO_2_ NP. However, we must notice that this second method is more time-consuming compared to the first one, and the tilt option from –25 to +25° must be available at the instrument used.

#### 4.1.3. Transmission Kikuchi Diffraction (TKD)

**Figure 10:**
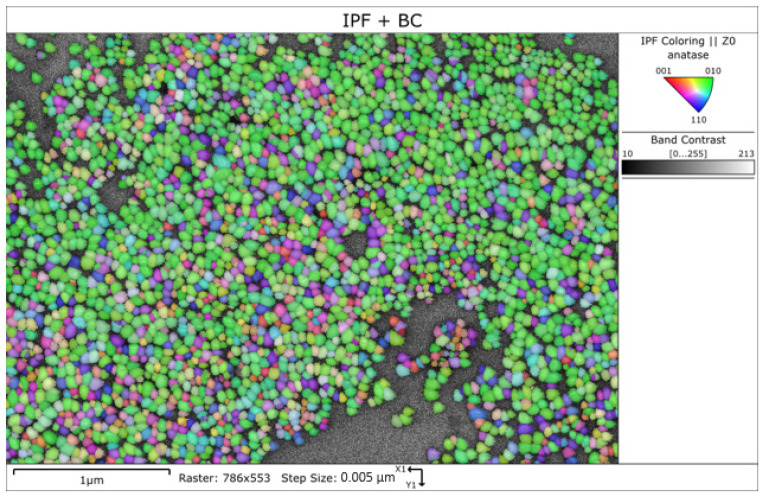
Inverse pole figure (IPF) with overlaid band contrast of TKD mapped TiO_2_ NPs.

An area densely covered with 2869 TiO_2_ NPs deposited on a carbon film TEM grid was mapped with TKD. The three possible geometrical orientations of the NPs are identified and displayed in Figure 10: ① lying on a {101} facet (blue), ② lying on the substrate with the NP long axis parallel to substrate (green), and ③ standing on a {001} facet (red). All three mentioned planes pole figures are presented in Figure S3. The angle between the {101} and {100} facets is defined by arctan of a/c, where a and c are lattice parameters of anatase (a = 3.7845 Å, c = 9.5143 Å), i.e., the angle is 21.69°. To differentiate unambiguously the two TiO_2_ NPs orientation variants ① and ②, data subsets were created by selecting grains with 10° threshold around {101} perfectly lying on carbon grid surface and by selecting grains with 10° threshold around {100} lying perfectly on the carbon grid surface. Such a threshold width was chosen to be approximately half of the 21.69° angle between the two TiO_2_ NPs orientation variants ① and ②.

A TKD map only with highlighted particles lying on a {101} facet is shown in Figure S4 together with the pole figures of the selected subset. A total number 474 TiO_2_ NPs are lying on a {101} facet (within 10° range around perfect {101} parallel to the carbon surface orientation) on the carbon film. The TKD map highlighting only those particles lying on a {100} facet is shown in Figure S5 together with the pole figures of the corresponding subset. A total number of 545 particles are lying on a {100} facet (within 10° range around the perfect {100} parallel to the carbon surface orientation). Very unlikely was the orientation ③ with only 6 TiO_2_ NPs of 2869 identified as lying on a {001} facet, see Figure S6.

Summing up the TKD analysis, one can conclude that the vast majority of the TiO_2_ NPs is arranged on the carbon film substrate in only two orientations, ① and ②, in roughly equal fractions; only an insignificant part lies in the orientation ③. Two observations are pertinent at this place: (i) an accurate NP size analysis by TKD based on the current measurement with a step size of 5 nm and a large spot size cannot be achieved, and (ii) the ratio of the two main NP geometrical orientations as found by TKD of nearly 1:1 for a carbon film substrate should not be implicitly the same as when the TiO_2_ NPs are deposited on a silicon substrate (as for the AFM and SE measurements, see later). In addition to the dependence of the particle orientation on the substrate type, there is also a dependence of the particle orientation on the degree of tightness of the NP as deposited on a substrate, i.e., the more agglomerated/entangled are the particles, the larger are the deviations from the two basic orientations ① and ②. The NPs from Figure 10 are mostly part of an incomplete monolayer.

### 4.2. Measurements of the Dimensional Parameters of TiO_2_ NPs

#### 4.2.1. Measurements of the Dimensional Parameters by Transmission Mode in SEM (STEM-in-SEM)

**Figure 11:**
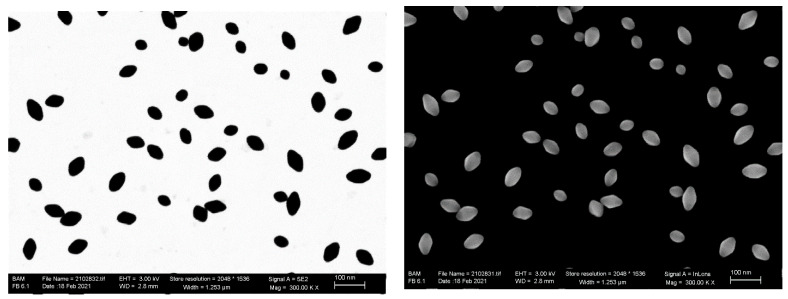
Three kilovolt STEM-in-SEM micrograph of the TiO_2_ NPs deposited on a carbon film TEM grid (**left**) and the corresponding 3 kV SEM (Zeiss-Supra40) secondary electrons InLens micrograph (**right**).

As described in the Section 2.5, STEM-in-SEM can be applied complementary to SEM on the same sample field-of-view and same NPs. An example is given in Figure 11.

The analysis of the 2D images in Figure 11 (both STEM-in-SEM and SEM) was carried out with the ImageJ software package [22], with selection of the ‘IsoData’ filter (which is the ‘Default’ setting) and by using a watershed algorithm for the segmentation thresholding. It should be noted that the vast majority of the TiO_2_ NPs are deposited on a carbon film TEM grid as isolated particles and the generally preferred orientation seems to be ② with the long axis of the NPs parallel with the substrate. A quantitative differentiation between the two possible geometrical orientations ① and ②, based only on the one pair of correlative STEM-in-SEM and SEM images as in Figure 10, is likely impossible. No other preferred orientation could be identified from the acquired images. Particularly in the STEM-in-SEM micrographs, the symmetry of practically all the particles imaged is obvious.

Two orientations cannot be identified from the 2D projection images, but *D*_fmin_ and *D*_fmax_ as mean values of the size distributions of same NPs with the two microscopy working modii, STEM-in-SEM and SEM, see Table 3.

Despite high-resolution images, a negative offset of about 5 nm has been found for the transmission case relative to the SEM mode. It is known that SEM, particularly in the InLens detection mode, overestimates the particles size due to (increased sensitivity) for edge effects (counting twice into the particle size due to two boundaries) [16]. This effect is diminished at low kV [23].

#### 4.2.2. Measurements of the Dimensional Parameters of Each NP Population of Different Geometrical Orientations by a Hybrid AFM/SEM Approach

A 350 TiO_2_ NPs population has been analyzed by hybrid metrology correlating AFM and another Zeiss-Ultra+ SEM. Once the orientation of each particle was determined by AFM, both microscopy techniques were implemented in order to measure the descriptors *D*_fmin_, *D*_fmax_ and *D_eq_* by SEM and the height of each NP, *h_AFM_*, by AFM.

The measurand, noted *D_eq_*, is the area-equivalent diameter extracted from the projected particle surface. This value corresponds to the diameter of a circle having the same surface area, *A*, as the measured particle:

$$D_{eq}=2\sqrt{\frac{A}{\pi }}$$

The various graphs representing the histograms of the size distributions of different the descriptors are included in Figure S7.

From a total of 350 measured TiO_2_ NPs, the following two fractions were determined: 75% NPs are oriented according to ① and the rest of 25% according to ②. A second observation is that the TiO_2_ NPs with orientations ① and ② are also different in their size distributions, i.e., of all descriptors *D_eq_*, *D*_fmin_ and *D*_fmax_. According to the results grouped together in the Table 4, it becomes evident that the two populations have distinct dimensions.

As shown in Section 4.1 (see Figure 5), the main characteristic parameters of oriented-② NPs can be directly determined by both Feret diameters. However, regarding oriented-① NPs, *D*_fmax_ measured from a 2D projection image does not correspond to the real particle length. The observed discrepancies between both populations could originate from these 2D projection effects.

The next section proposes an approach how to measure accurately the true external dimensions of the oriented-① TiO_2_ NPs, particularly of *D*_fmax_.

The comparison between the results obtained with whole population and reported in Table 3 and Table 4 shows SEM (Zeiss-Ultra+ and Zeiss-Supra40) measurements are this time very close to those obtained with STEM-in-SEM. However, we must keep in mind that the behavior of nanoparticles deposited on carbon TEM-grid and silicon wafer can be different, leading to different ratios between ① and ② oriented populations. The Figure S8 shows the consistency of measurement results between Zeiss-Supra40 SEM, Zeiss-Supra40 STEM-in-SEM, Zeiss-Ultra+ SEM as well as between *D*_Fmin_ and *h_AFM_.* The uncertainties associated with measurements are also reported in the graphs.

### 4.3. Measurements of the True Size Descriptors, L and s, of the Population Oriented ①

**Figure 12:**
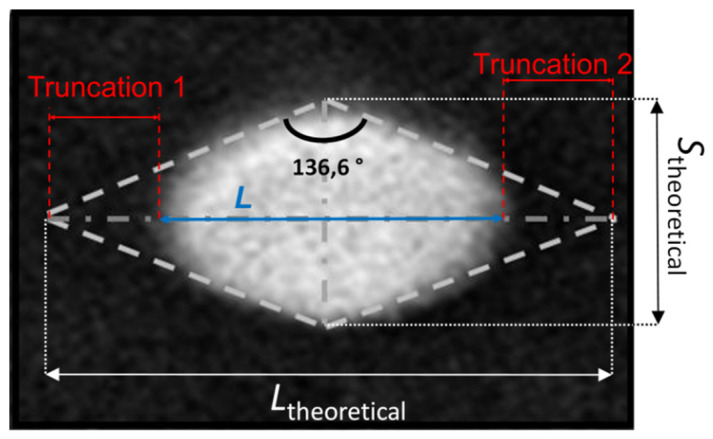
Example of a TiO_2_ NP oriented ② as imaged with SEM with an illustration of the expected theoretical bipyramidal structure marked in dashed lines.

As shown in Figure 12, the synthesized TiO_2_ NPs are truncated. The dashed lines delimit the theoretical shape of these particles as they were untruncated. On both sides, the peaks are missing because, from a thermodynamic point of view, the energy cost is too high to complete the structure. However, between two adjacent {101} faces, an angle close to the theoretical value of 136.6° was measured on all the particles studied as shown in Figure 8. In the case of a perfect bipyramid with an angle of 136.6°, the expected TiO_2_ NPs aspect ratio (i.e., *L*_theoretical_ / *s*_theoretical_ = tan(136.6°/2)) is 2.51.

**Figure 13:**
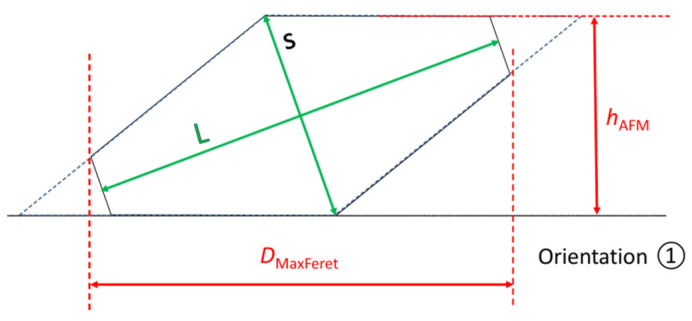
Schematic view of a truncated TiO_2_ NPs oriented ①, with various descriptors annotated.

Regarding the TiO_2_ NPs oriented ① (see Figure 13), too many parameters are unknown to accurately determine the value of *L*. We have no way of measuring the exact orientation of NPs (*α* angle) by only SEM and determine the dimensions of the truncated part of each peak. Further, the largest measurement uncertainty in setting the SEM segmentation threshold over the entire particle boundary is at the peaks of the bipyramid (biggest edge effects). Nevertheless, as specified above, the parameter *s* can be directly measured for the TiO_2_ NPs oriented ① by means of *D*_fmin_.

For the TiO_2_ NPs oriented ① the parameter *L* is determined from the maximum value of measured *D*_fmax_ as a function of the tilting angle, i.e., the longest *D*_fmax_ can be found at that tilt at which the long axis of the particle becomes perpendicular to the electron beam. In this way, the parameters *L* of around 50 TiO_2_ NPs were determined by tilting the stage. The error made by a direct *D_MaxFeret_* measurement at 0° has been estimated. The relative deviation found between this maximum value and the *D*_fmax_ value at 0° has been found to be 3.6% ± 1.7% on average. This difference is tiny; however, the approach demonstrates its applicability if appropriately applied on a representative number of NPs.

Further, the experimentally found difference of 3.6% in *D*_fmax_ by tilting was used to (re-)calculate the true parameter *L* of the TiO_2_ NPs oriented ① regarding the complete population of 350 TiO_2_ NPs. Together with the results of the NPs oriented ② they are reported in Table 5. The results given by this comparative table clearly conclude that the two populations have a different morphology. They confirm that TiO_2_ NPs oriented ① are larger and have a higher aspect ratio than the NPs orientated ② (1.49 versus 1.37). The population ② is more equiaxial.

### 4.4. Evaluation of the Size of the TiO_2_ NPs Truncations

The dimensions of the truncations were evaluated from the TiO_2_ NPs imaged by SEM (Figure 12). With this challenging parameter, it is possible to obtain further information on the true TiO_2_ NPs geometry. This geometry could then be used to be included a-priori for fine particle size analysis by SAXS. To evaluate the dimensions of the bipyramid truncations, a Matlab routine was developed. This routine was used to fit the theoretical shape of the perfect particle on the bipyramid contours. By calculating, for each particle, the difference between the theoretical length of the perfectly shaped bipyramid and the imaged one, the dimension of the truncations can be evaluated on a large number of TiO_2_ NPs. Results are presented in Table 6 for NPs according to their two main orientations ① and ②.

The results show that the size of the truncations is significant. This proves that the measured aspect ratios, which are very different from the theoretical ones, are related to a significant truncation of the particles along their major axis.

Finally, the actual size, shape and geometrical orientation of the TiO_2_ NPs can be accurately described thanks to the AFM/SEM hybrid approach. Most of the TiO_2_ NPs are lying on the substrate on one of their {101} face. The TiO_2_ NPs have a much lower aspect ratio (of 1.37 to 1.49) than the theoretical one (2.51), because of very pronounced truncations along their long axis. Truncations are also present along their minor axis, however, these have a minor influence on the size measurement.

### 4.5. Comparison with an Ensemble Technique: SAXS

**Figure 14:**
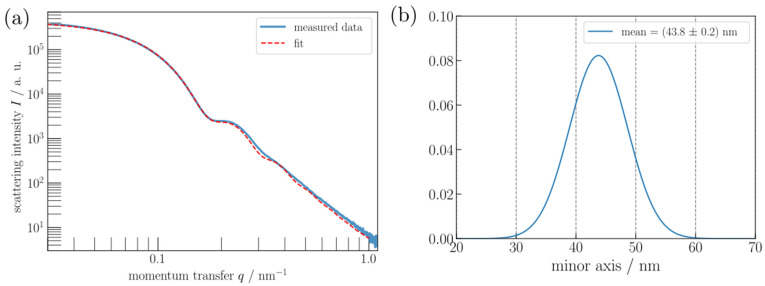
(**a**) SAXS: Fit of the measured data of the TiO_2_ bipyramids assuming the aspect ratio of the truncated bipyramids given in Table 4 for orientation ②. (**b**) Number-weighted distribution of the minor axis of the TiO_2_ bipyramids.

For the evaluation of the SAXS measurements, thanks to the results previously obtained by microscopy, we assumed a truncated bipyramidal particle shape which was constructed from the dimensional values of orientation ② given in Table 5 and Table 6. This shape was then filled quasi-randomly with 30,000 punctiform scatterers with uniform density. Thereafter, Debye’s scattering equation is applied onto the cloud of evenly distributed scatterers to retrieve the numerically computed form factor [19]. With this form factor and assuming a Gaussian size distribution of the particles, the measured scattering curve was then fitted using a Markov-Chain-Monte-Carlo-algorithm according to Foreman-Mackey et al. [24] revealing an uncertainty estimate for mean value of the minor axis and the standard deviation of the corresponding distribution width. Only the particle size is changed during the fitting procedure whereas the shape is kept constant. The fit results in a mean value of the minor axis (edge length) of the truncated bipyramidal model of (43.8 ± 0.2) nm with a standard deviation of the size distribution of (4.8 ± 0.2) nm (Figure 14). This uncertainty is certainly underestimated because the shape was assumed to be constant. Additionally, the model curve does not fit the measured data perfectly (Figure 14), as it displays more discernible minima. This can be caused by different shapes of the size distribution as well as a distribution of the shape, rather than the size of the particles alone. Based on the assumed constant ratio between minor and major axis of the truncated bipyramid in orientation 2, the mean value of the major axis is 60.1 nm. We have also varied the aspect ratio, i.e., the ratio between major and minor axis of the bipyramid shape, to study the sensitivity of the SAXS measurement to the particle shape. It turns out that the form factor is influenced mostly by the minor axis, i.e., the side length of the pyramid base, and nearly independent of the major axis. Thus, the minor axis can be determined more precisely from the SAXS measurement.

Thus, taking into account the measurement uncertainties obtained by SEM, AFM and STEM-in-SEM, the results on the size of bipyramids measured by SAXS with the sample in liquid suspension are consistent with those obtained by microscopy (Table 5) with the NPs deposited on a substrate.

In order to determine the number concentration of the NPs from SAXS, the electron density contrast additionally needs to be known [18]. We used a value of 890 nm^−3^ for TiO_2_ in water at a photon energy of E_ph_ = 8 keV which was calculated from an assumed bulk density of 4.24 g cm^−3^. The energy-dependence of the electron contrast was taken into account by replacing the atomic numbers Z of titanium and oxygen by the corresponding energy-depending atomic form factors f_1_(E_ph_) + i f_2_(E_ph_).

This results in a number concentration a value of 1.2.10^13^ particles per mL.

## 5. Conclusions

In this study, we demonstrated the feasibility of a correlative analysis approach based on EM and AFM on one hand, and TKD and SAXS on the other hand, to obtain a comprehensive information of the dimensions and shape of a NP population of complex shape.

First, we demonstrated the relevance of implementing hybrid metrology correlating AFM and EM to characterize in 3D a population of nanoparticles with complex shape (bipyramids). The dimensional properties of nanosized TiO_2_ NPs bipyramids were measured and two descriptors, *L* and *s*, were determined corresponding to length and squared base side, respectively. Several experimental approaches have been used: (i) AFM profiling along the long axis of the TiO_2_ NPs, (ii) sequential tilting and SEM imaging with the extraction of the longest *D*_fmax_ as a descriptor characterizing the true length of the TiO_2_ NPs, independent of their geometric orientation on the substrate and (iii) TKD analysis to obtain information on the geometrical orientation of a greater number of deposited particles. It should be noticed that the type of substrate and sample deposition (quality and NP density) may significantly influence the exact orientation of the NPs on the substrate. An analysis of the same NPs prepared on the same substrate with all the techniques could not be achieved, only pairs of two methods are currently possible to be correlated.

We could successfully determine the following parameters: (i) the geometrical orientation of each nanoparticle as deposited on the substrate, (ii) the exact angle between two adjacent {101} faces, and (iii) the main characteristic parameters, *L*, *s* and the aspect ratio. Particularly for the accurate determination of *L*, the truncation of the bipyramids has been measured. Two sub-populations have been found by the systematic AFM-EM hybrid approach; one population constitutes of larger particles lying with a {101} face on the silicon substrate, and the other NP fraction rather balancing on the substrate along their long axis.

Finally, as these characteristic parameters, evaluated on a limited number of NP, are available, we are able to feed a specific SAXS model with the aspect ratio measured by microscopy. From this model, a comprehensive information on the dimensional parameters of the TiO_2_ bi-pyramids was obtained. The SAXS results were consistent with those provided by the microscopy methods. Further, this study highlights that by obtaining information on the shape of a small number of particles by microscopy, we can obtain reliable and accurate integral information by SAXS.

## Figures and Tables

**Table 1:** Substrates and deposition method used according to the microscopy techniques.

Method	Substrate	Functionalization of the Substrate	Deposition Method
SEM—AFM	Silicon chip	Yes with PLL	Spin Coating
STEM-in-SEM—TKD	Carbon TEM grid	No	Spin coating

**Table 2:** Setup parameters used for SEM imaging.

Parameter	Value
Accelerating voltage	3 kV
Working distance	3 mm
Magnification	×40,000
Image resolution	2048 × 1536 pixels
Pixel size	1.4 nm
Time frame for image acquisition	28.4 s

**Table 3:** Result of the image analysis of the same field-of-view with TiO_2_ NPs by Zeiss-Supra40 SEM with InLens secondary electron detection and by Zeiss-Supra 40 STEM-in-SEM, both at 3 kV.

Microscopy Mode	Counted Particles	*D*_fmin_/nm	*D*_fmax_/nm	Aspect Ratio
Zeiss-Supra40 SEM	102	48.2 ± 5.7	69.3 ± 13.4	1.42 ± 0.17
Zeiss-Supra40 STEM-in-SEM	103	43.5 ± 5.6	64.8 ± 13.2	1.47 ± 0.19

**Table 4:** Different descriptors as determined for each population of TiO_2_ NPs.

	Whole Population	Orientation ①	Orientation ②
*D_eq_* (nm)	51.2 ± 1.7	51.9 ± 1.7	48.9 ± 1.8
*D*_fmax_ (nm)	63.2 ± 1.8	64.4 ± 1.8	59.6 ± 2.0
*D*_fmin_ (nm)	44.9 ± 1.7	45.4 ± 1.7	43.4 ± 1.8
R (aspect ratio)	1.40 ± 0.07	1.41 ± 0.07	1.37 ± 0.07
*h_AFM_* (nm)	42.4 ± 0.6	42.8 ± 0.6	41.2 ± 0.7

**Table 5:** Size and shape results obtained for the two populations oriented ① and ②.

	Orientation ①	Orientation ②
*L* (nm)	68.3 ± 2.1	59.6 ± 2.3
*s* (nm)	45.9 ± 1.9	43.4 ± 1.9
*R* (aspect ratio)	1.49 ± 0.08	1.37 ± 0.08

**Table 6:** Length of the NP truncations (at one side only) along their major axis according to the TiO_2_ NPs orientation.

TiO_2_ NPs	Length of the Truncations/nm
Orientation ①	31.8 ± 3.1
Orientation ②	31.8 ± 5.0

## Data Availability

Not applicable.

## Supplementary Materials

The following are available online at www.mdpi.com/article/10.3390/nano11123359/s1, Figure S1: STEM-in-SEM image, taken at 20 kV, of the particles shown in Figure 3; Figure S2: (**a**) Transmission Kikuchi diffraction pattern of anatase bipyramid nanoparticles and (**b**) indexed pattern with the Aztec acquisition software; Figure S3: Pole figures of {101}, {100}, and {001} from the map presented in Figure 9; Figure S4: Highlighted particles lying on a {100} facet and their pole figures; Figure S5: Highlighted particles lying on a {101} facet and their pole figures; Figure S6: Highlighted particles lying on a {001} facet and their pole figures; Figure S7: Dimensional parameters Deq, Dfmin and Dfmax as measured by Zeiss-Ultra+ SEM for each NP population fraction with the orientations ① and ② from a total of 350 particles. The bin size of histograms depends on the number of nanoparticles involved; Figure S8: Results of Dfmin, Dfmax and Dfmin/Dfmax with their associated uncertainties for the Zeiss-Supra40 SEM, Zeiss-Supra40 STEM-in-SEM and Zeiss-Ultra+ SEM. The hAFM measurements are compared to the Dfmin results in the figure on the left.
