# Supplementary material for: Correlative Analysis of the Dimensional Properties of Bipyramidal Titania Nanoparticles by Complementing Electron Microscopy with Other Methods
Source: Nanomaterials (Basel). 2021 Dec 10;11(12):3359. doi: 10.3390/nano11123359 (PMC8708744; doi:10.3390/nano11123359)
Supplement: Supplementary file 1 [file nanomaterials-11-03359-s001.zip › nanomaterials-1423305-supplementary.pdf]

nanomaterials

Supplementary Information

Correlative Analysis of the Dimensional Properties of Bipyramidal Titania Nanoparticles by Complementing Electron Microscopy with Other Methods

Loïc Crouzier [1,*], Nicolas Feltin [1], Alexandra Delvallée [1], Francesco Pellegrino [2], Valter Maurino [2], Grzegorz Cios [3], Tomasz Tokarski [3], Christoph Salzmann [4], Jerome Deumer [5], Christian Gollwitzer [5] and Vasile-Dan Hodoroaba [4]

1 Laboratoire National de métrologie et d'Essais (LNE), 29 Avenue Roger Hennequin, CEDEX, 78197 Trappes, France; nicolas.feltin@lne.fr (N.F.); Alexandra.Delvallee@lne.fr (A.D.)
2 Dipartimento di Chimica and NIS Inter-Department Centre, University of Torino, Via P. Giuria 7, 10125 Torino, Italy; francesco.pellegrino@unito.it (F.P.); valter.maurino@unito.it (V.M.)
3 Academic Centre for Materials and Nanotechnology, AGH University of Science and Technology, Mickiewicza 30, 30-059 Krakow, Poland; ciosu@agh.edu.pl (G.C.); tokarski@agh.edu.pl (T.T.)
4 Federal Institute for Materials Research and Testing (BAM), Unter den Eichen 44-46, 12205 Berlin, Germany; ch.salzmann@web.de (C.S.); dan.hodoroaba@bam.de (V.-D.H.)
5 Physikalisch-Technische Bundesanstalt (PTB), Abbestraße 2–12, 10587 Berlin, Germany; jerome.deumer@ptb.de (J.D.);christian.gollwitzer@ptb.de (C.G.)
* Correspondence: Loic.Crouzier@lne.fr

Citation: Crouzier, L.; Feltin, N.; Delvallée, A.; Pellegrino, F.; Maurino, V.; Cios, G.; Tokarski, T.; Salzmann, C.; Deumer, J.; Gollwitzer, C.; Hodoroaba, V.-D.; Correlative Analysis of the Dimensional Properties of Bipyramidal Titania Nanoparticles by Complementing Electron Microscopy with Other Methods. *Nanomaterials* **2021**, *11*, 3359. https://doi.org/10.3390/nano11123359

Academic Editor: David Marrero-López

Received: 30 September 2021
Accepted: 8 December 2021
Published: 10 December 2021

Publisher's Note: MDPI stays neutral with regard to jurisdictional claims in published maps and institutional affiliations.

Copyright: © 2021 by the author. Licensee MDPI, Basel, Switzerland. This article is an open access article distributed under the terms and conditions of the Creative Commons Attribution (CC BY) license (https://creativecommons.org/licenses/by/4.0/).

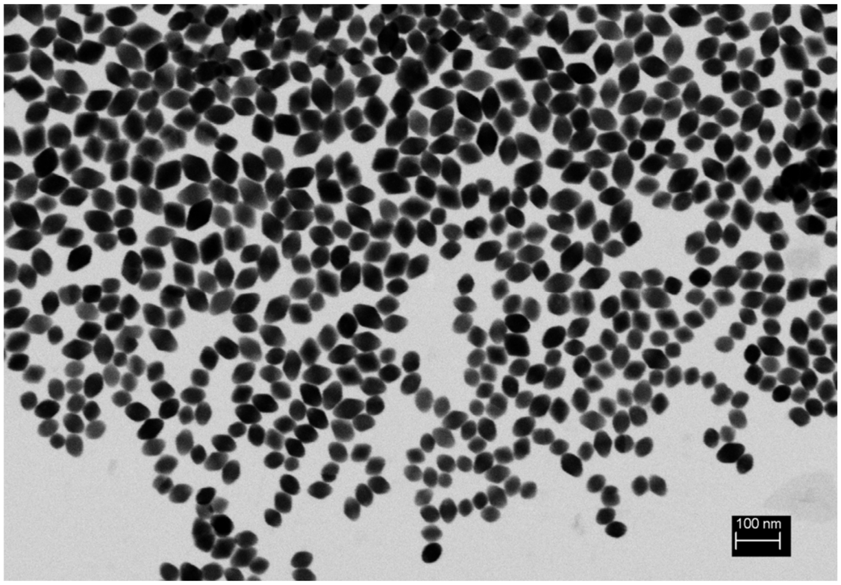

Figure S1. STEM-in-SEM image, taken at 20 kV, of the particles shown in Figure 3.

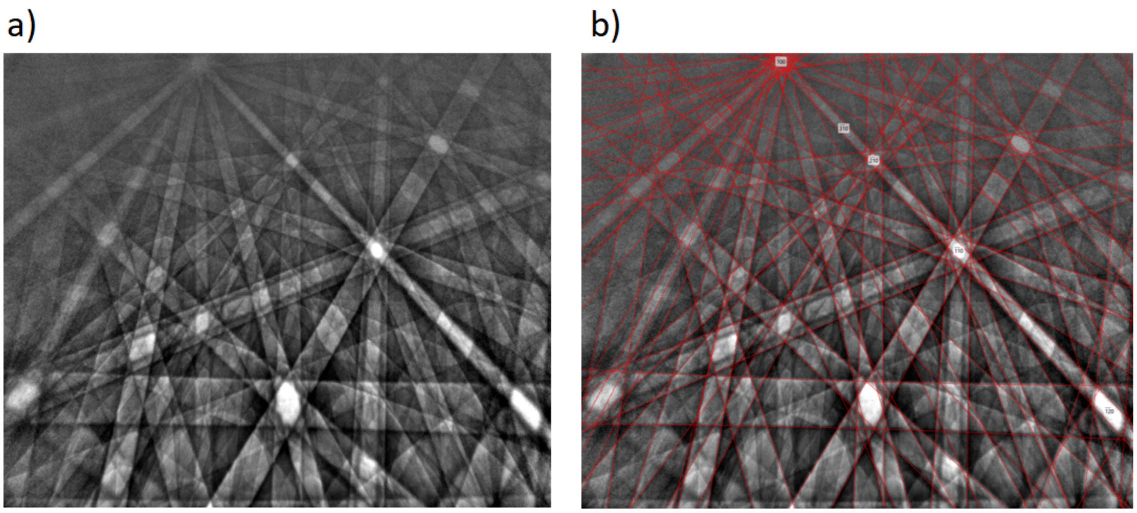

Figure S2. (**a**) Transmission Kikuchi diffraction pattern of anatase bipyramid nanoparticles and (**b**) indexed pattern with the Aztec acquisition software.

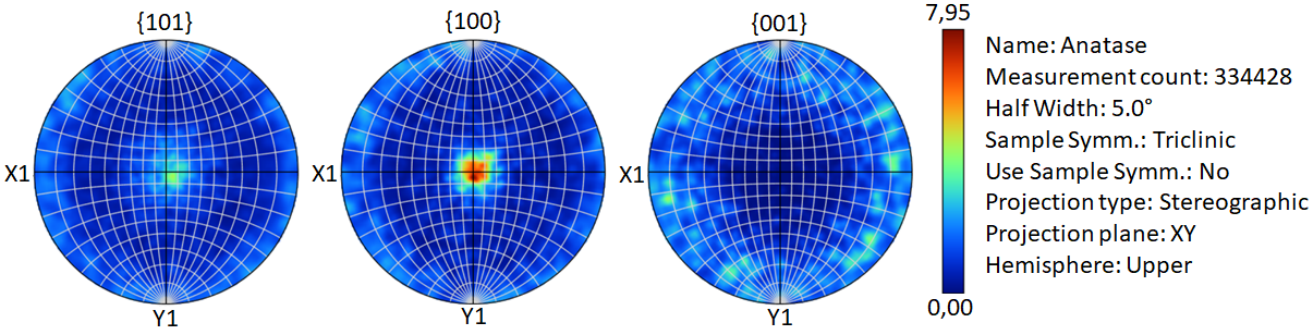

Figure S3. Pole figures of {101}, {100}, and {001} from the map presented in Figure 9.

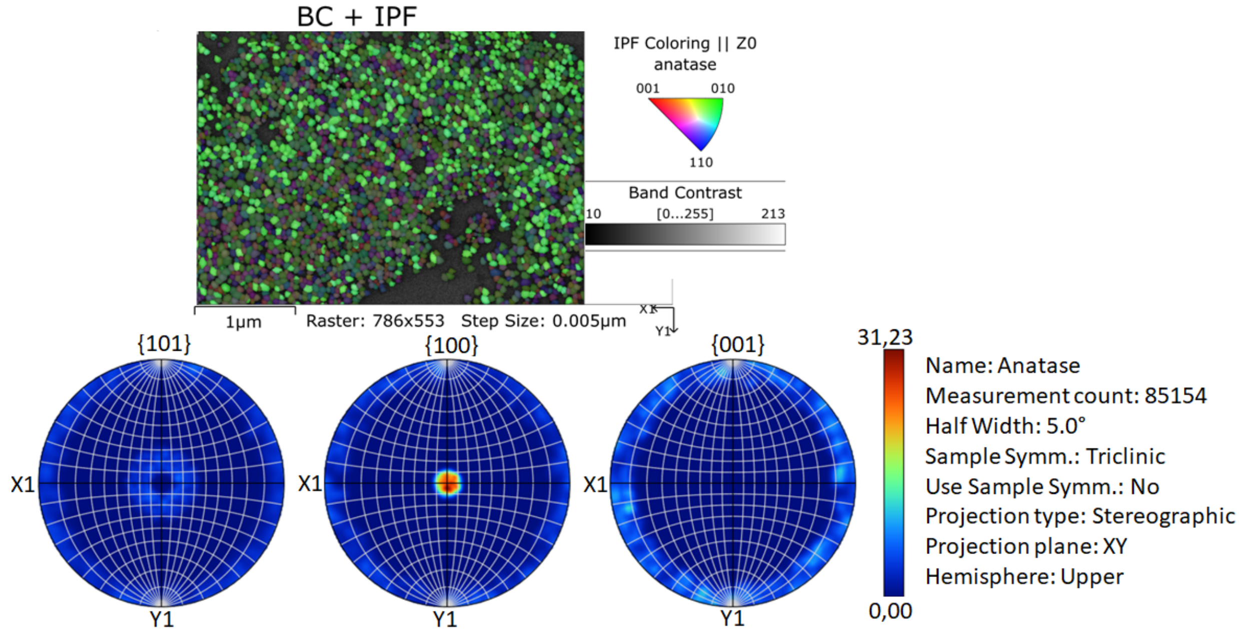

Figure S4. Highlighted particles lying on a {100} facet and their pole figures.

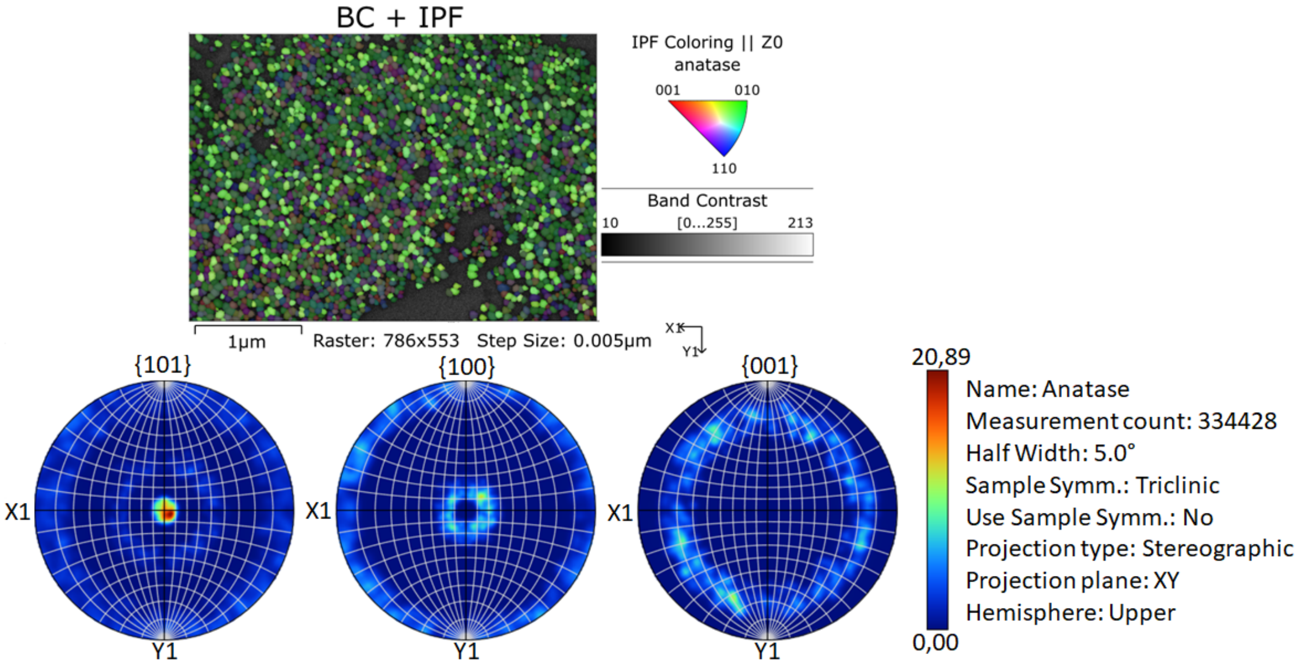

Figure S5. Highlighted particles lying on a {101} facet and their pole figures.

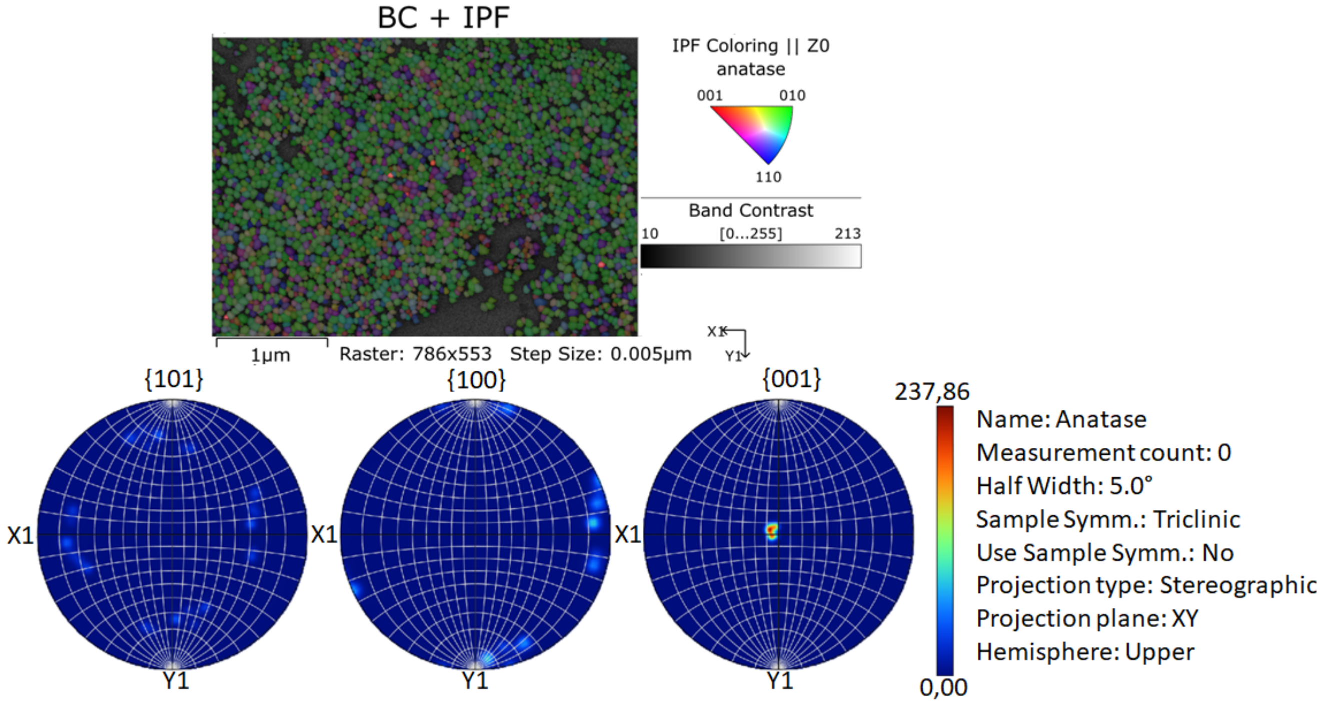

Figure S6. Highlighted particles lying on a {001} facet and their pole figures.

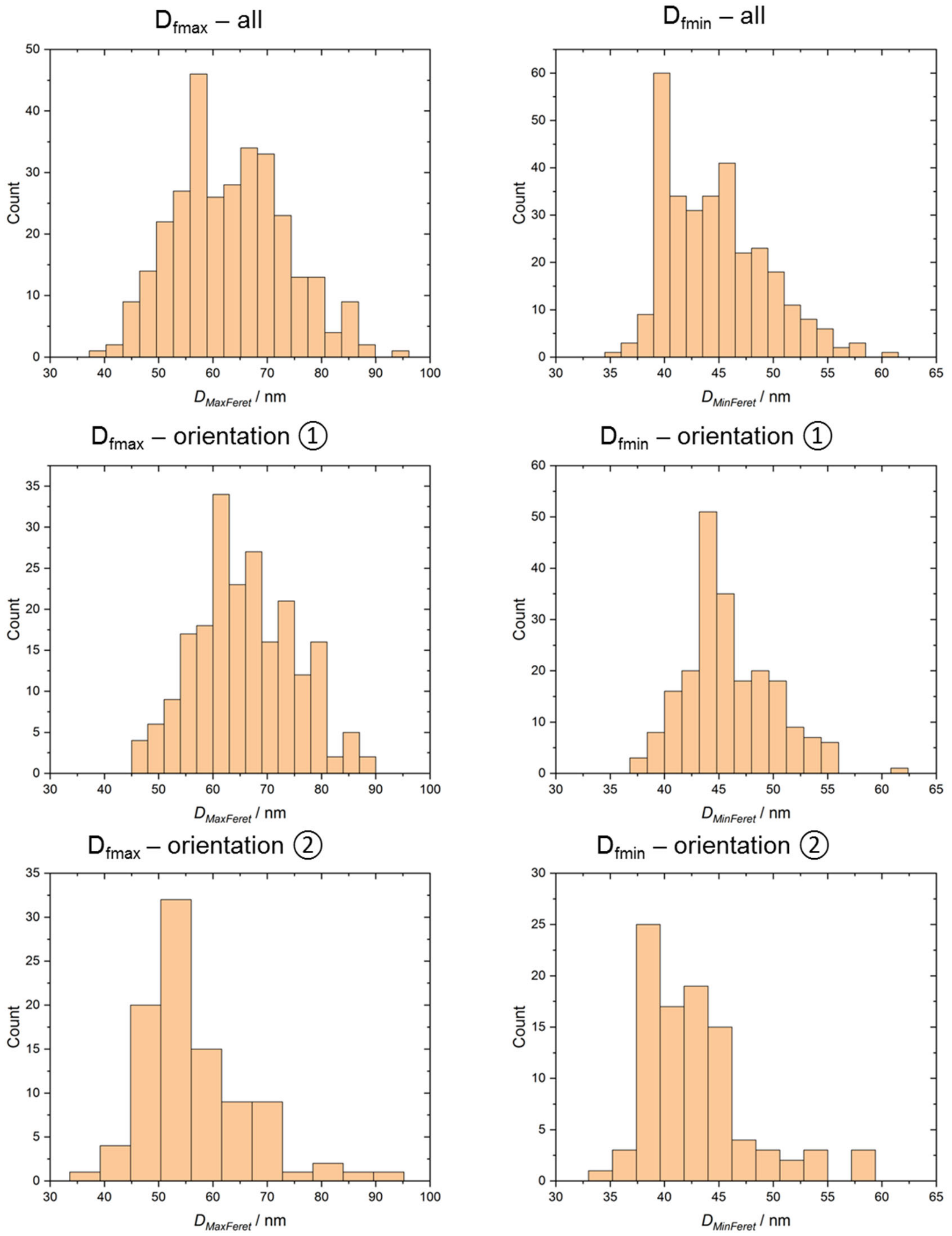

Figure S7. Dimensional parameters D_{eq}, D_{fmin} and D_{fmax} as measured by Zeiss-Ultra+ SEM for each NP population fraction with the orientations ① and ② from a total of 350 particles. The bin size of histograms depends on the number of nanoparticles involved.

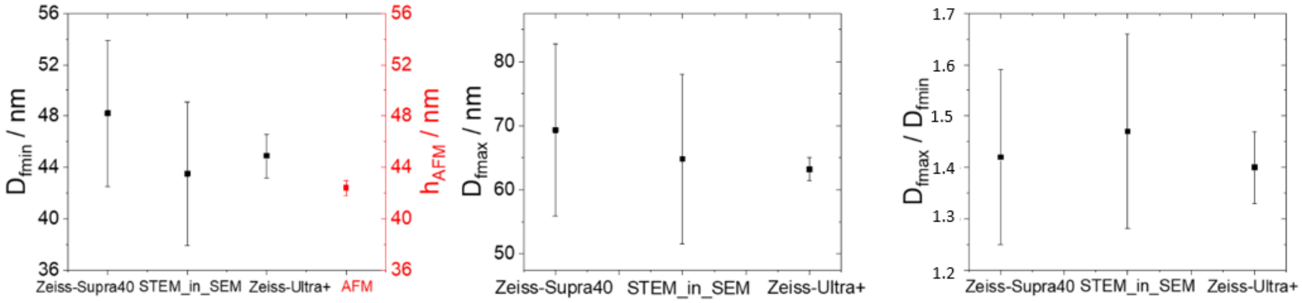

Figure S8. Results of D_{fmin}, D_{fmax} and D_{fmin}/D_{fmax} with their associated uncertainties for the Zeiss-Supra40 SEM, Zeiss-Supra40 STEM-in-SEM and Zeiss-Ultra+ SEM. The h_{AFM} measurements are compared to the D_{fmin} results in the figure on the left.
